# Comparative Diagnostic Efficacy of Four Breast Imaging Modalities in Dense Breasts: A Single-Center Retrospective Study

**DOI:** 10.3390/biomedicines13071750

**Published:** 2025-07-17

**Authors:** Danka Petrović, Bojana Šćepanović, Milena Spirovski, Zoran Nikin, Nataša Prvulović Bunović

**Affiliations:** 1 Department of Radiological Diagnostics, Oncology Institute of Vojvodina, 21204 Sremska Kamenica, Serbia; drbojanascepanovic@gmail.com (B.Š.); milena.spirovski@mf.uns.ac.rs (M.S.); natasa.prvulovic-bunovic@mf.uns.ac.rs (N.P.B.); 2 Department of Oncology, Faculty of Medicine, University of Novi Sad, 21000 Novi Sad, Serbia; 3 Department of Pathology, Oncology Institute of Vojvodina, 21204 Sremska Kamenica, Serbia; nikinzoran@gmail.com; 4 Department of Nuclear Medicine, Faculty of Medicine, University of Novi Sad, 21000 Novi Sad, Serbia

**Keywords:** dense breasts, diagnostic imaging, digital mammography, digital breast tomosynthesis, ultrasound, breast MRI, sensitivity, specificity, diagnostic accuracy

## Abstract

**Background and Objectives**: The aim of our study was to assess the diagnostic accuracy of four imaging modalities—digital mammography (DM), digital breast tomosynthesis (DBT), ultrasound (US), and breast magnetic resonance imaging (MRI)—applied individually and in combination in early cancer detection in women with dense breasts. **Methods**: This single-center retrospective study was conducted from January 2021 to September 2024 at the Oncology Institute of Vojvodina in Serbia and included 168 asymptomatic and symptomatic women with dense breasts. Based on the exclusion criteria, the final number of women who were screened with all four imaging methods was 156. The reference standard for checking the diagnostic accuracy of these methods is the result of a histopathological examination, if a biopsy is performed, or a stable radiological finding in the next 12–24 months. **Results**: The findings underscore the superior diagnostic performance of breast MRI with the highest sensitivity (95.1%), specificity (78.7%), and overall accuracy (87.2%). In contrast, DM showed the lowest sensitivity (87.7%) and low specificity (49.3%). While the combination of DM + DBT + US demonstrated improved sensitivity to 96.3%, its specificity drastically decreased to 32%, illustrating as ensitivity–specificity trade-off. Notably, the integration of all four modalities increased sensitivity to 97.5% but decreased specificity to 29.3%, suggesting an overdiagnosis risk. DBT significantly improved performance over DM alone, likely due to enhanced tissue differentiation. US proved valuable in dense breast tissue but was associated with a high false-positive rate. Breast MRI, even when used alone, confirmed its status as the gold standard for dense breast imaging. However, its widespread use is constrained by economic and logistical barriers. ROC curve analysis further emphasized MRI’s diagnostic superiority (AUC = 0.958) compared with US (0.863), DBT (0.828), and DM (0.820). **Conclusions**: This study provides a unique, comprehensive comparison of all four imaging modalities within the same patient cohort, offering a rare model for optimizing diagnostic pathways in women with dense breasts. The findings support the strategic integration of complementary imaging approaches to improve early cancer detection while highlighting the risk of increased false-positive rates. In settings where MRI is not readily accessible, a combined DM + DBT + US protocol may serve as a pragmatic alternative, though its limitations in specificity must be carefully considered.

## 1. Introduction

Breast cancer is the most commonly diagnosed female malignancy and the main cause of cancer deaths in women worldwide [1]. According to the latest available data from the World Health Organization and estimates from the International Agency for Research on Cancer, in 2022, the incidence rate was 46.8 per 100,000, while the mortality rate was 12.7 per 100,000 in women of all ages worldwide [2]. It is a significant global health concern, demanding improved and effective imaging algorithms for the early detection of breast cancer for reducing breast cancer-related mortality, especially in dense breasts [2,3,4].

Dense breasts are one of the primary risk factors for breast cancer, close to age and BRCA1/2 mutations [5]. Approximately 50% of women have dense breast tissue [6]. This condition refers to a mammographic composition characterized by a higher proportion of fibro glandular tissue relative to fatty tissue. This denser composition appears white on mammographic images, the same density as many tumors, which can obscure the presence of small, non-calcified tumors [7]. According to the 5th edition of the ACR BI-RADS (American College of Radiology Breast Imaging Reporting and Data System) atlas, breast density is categorized into heterogeneously dense (ACR C) and extremely dense (ACR D) types [8]. Subjective visual estimation by radiologist remains widely used in clinical practice across many countries, despite advances in automated density measurement tools [9]. Data from the Breast Cancer Surveillance Consortium (BCSC) indicate that dense breasts contribute to a population-attributable risk of invasive breast cancer ranging from 24% to 35%, compared with13% to 17% in women with fatty breasts [10]. Women with extremely dense breasts account for10% of the female population and carry a significantly higher risk for delayed breast cancer diagnoses, with interval cancers occurring up to 18 times more frequently than in women with fatty breasts [11]. The literature indicates that the sensitivity of digital mammography (DM) in women with extremely dense breasts ranges from approximately 57% to 71%, underlining that this population is inadequately served by DM alone, which is widely regarded as the gold standard for breast cancer screening in women at average risk [12,13,14]. Consequently, several developed countries have enacted legislation requiring that women be informed by radiologists about their breast density and the potential need for supplemental screening [15].

Emerging strategies in breast cancer imaging are refining diagnostic accuracy for women with dense breast tissue, where conventional mammography has limited sensitivity. A comprehensive review proposes the progressive integration of digital breast tomosynthesis (DBT), ultrasound (US), and breast MRI, emphasizing the need for the evidence-based selection and combination of modalities to optimize specificity and sensitivity depending on individual clinical factors [16].

DBT represents a significant advancement in breast imaging, offering enhanced detection capabilities and complementing DM [17]. Its widespread adoption will depend on overcoming infrastructure and training challenges, while ongoing research and development promise to expand its applications to breast screening [18,19]. DBT is an advanced X-ray imaging breast technique which employs multiple low-dose X-rays taken at various angles around the breast and then use computer software to synthesize these into a three-dimensional (3D) image [20,21]. It is designed to eliminate overlapping and summation effects of fibro glandular structures inherent to DM and improve the detection and characterization of small non-calcified lesions by enhancing lesion margin clarity and reducing the number of false-negative and false-positive findings in dense breasts [22]. However, DBT still relies on ionizing radiation, albeit with optimized doses in synthesized mammography combinations. The synthetic DM images exported from DBT can replace conventional DM images and reduce the radiation dose on breasts by 30 to 40 percent compared with DBT plus conventional DM screening [23].

US has emerged as a critical adjunct to DM in the detection of breast cancer in women with dense breast tissue [24]. A systematic review underscores that while DM remains basic, its efficacy diminishes in dense breasts, prompting the integration of US, DBT, and breast MRI to enhance diagnostic precision [25,26,27]. In contrast, US is a radiation-free, accessible modality that improves cancer detection in dense breast tissue, especially when used adjunctively to DM + DBT, and thereby reduces the number of interval cancers [28]. But the limitation sar operator dependence and the equipment used. Automated breast ultrasound (ABUS) seeks to address these limitations but still faces technological constraints [29]. Different medical institutions have established guidelines recommending that women with dense breast tissue consider additional imaging modalities, such as US [30]. These recommendations are designed to improve cancer detection rates by utilizing the most effective tools tailored to individual conditions. In summary, ultrasound serves as a valuable adjunct in the assessment of dense breasts by providing enhanced detection capabilities and complementing DM [28].

The literature indicates that breast MRI, due to the use of contrast agents, achieves the highest sensitivity and specificity among imaging modalities, significantly reducing false-negative findings and mortality from advanced disease [31]. It remains the standard technique for assessing tumor neo angiogenesis, improving both the detection and characterization of malignant lesions [32]. Breast MRI is also associated with lower interval cancer rates, supporting its use in screening protocols for high-risk women [33]. Additional benefits of MRI include reduced radiation exposure, the use of gadolinium-based contrast agents with fewer side effects than iodine-based agents, and the ability to concurrently evaluate axillary lymph node metastases [34]. According to the European Society of Breast Imaging (EUSOBI), breast MRI is recommended as supplemental screening for women aged 50 to 70 years with extremely dense breast tissue, to be performed once every 2 to 4years [35]. However, many countries lack the necessary infrastructure and MRI unit availability to implement this recommendation on a population-wide scale. Nonetheless, MRI is limited by high costs, prolonged acquisition times, and restricted accessibility, and it often lacks sensitivity in detecting ductal carcinoma in situ (DCIS), even in high-grade cases.

A multimodal, risk-adapted screening approach that considers individual patient risk, breast density, and local resource availability remains the most effective strategy for optimizing early breast cancer detection and minimizing mortality [27]. These findings support an optimized supplemental imaging approach to breast cancer detection in dense breasts, blending technological advancements with professional proficiency to improve early cancer detection while minimizing false positives and patient burden like increased image interpretation time, patient anxiety, and radiation dose. Reduced recalled rates at screening with a multimodal protocol can reduce unnecessary biopsies of benign lesions, overdiagnosis, and associated risks and costs [36]. There are variations in assessment protocols across different countries which must be reduced to standardize care for women recalled at screening.

The aim of our study was to determine the diagnostic accuracy of DM, DBT, US, and breast MRI both individually and in combination in early cancer detection in women with dense breasts. While several studies in the literature have compared subsets of these imaging modalities (e.g., DM vs. DBT, DM + DBT vs. US, or MRI vs. DM/DBT), we found no published studies to date that directly and simultaneously compared all four imaging methods applied within the same cohort and diagnostic workup pathway, particularly in the context of dense breast tissue. We also included a brief literature synthesis citing key comparative studies that addressed subsets of these modalities (e.g., Raich and et al., 2024, Akwo et al., 2024, Abu Abeeleh et al., 2024) [18,27,37]. This contextualizes our study as an extension that fills an important gap in comprehensive multimodal evaluation.

## 2. Materials and Methods

### 2.1. Study Design and Patient Selection

Our clinical research was conducted as a cross-sectional retrospective study at the Department of Radiological Diagnostics, Oncology Institute of Vojvodina (IOV), during the period from January 2021 to September 2024. The study was approved by the Institutional Ethics Committee. During this period, a total of 12,149 women—both asymptomatic and symptomatic—were examined based on appropriate breast imaging at our institution. Out of the total number of examined women, a sample of 168 women were found to have dense breasts on DM, categorized as ACR C or D, and underwent all four breast imaging modalities: DBT performed concurrently with DM, followed by US and breast MRI within a twelve-week interval.

Based on the exclusion criteria, 12 patients were excluded from the study, resulting in a final cohort of 156 women. Needle or surgical biopsies were performed for all suspicious findings classified as BI-RADS 4 and 5, based on the BI-RADS atlas. Cases categorized as BI-RADS 1, 2, or 3 were followed up using appropriate radiological methods over a 12- to 24-month period; those that remained radiological stable were considered benign at the end of the follow-up period. Histopathology examination (HPE) conducted at the Department of Histopathology, Oncology Institute of Vojvodina, was the gold standard for evaluating the diagnostic accuracy of all four imaging modalities.

**Figure 1:**
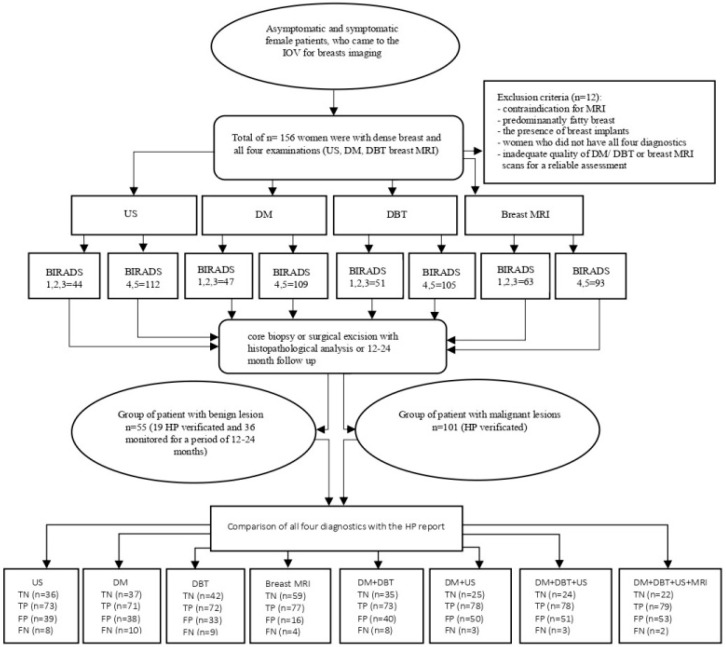
The general flowchart shows the methodological steps of the study. Abbreviations: US: ultrasound; DM: digital mammography; DBT: digital breast tomosynthesis; breast MRI: breast magnetic resonance imaging; BI-RADS: Breast Imaging Reporting and Data System; HPE: histopathology; TN: true negative; TP: true positive; FP: false positive; FN: false negative.

The exclusion criteria included the presence of breast implants and inadequate image quality of DM/DBT or breast MRI rendering reliable assessment impossible. The following flowchart (Figure 1) illustrates the methodological steps of the study, including patient selection, diagnostic imaging procedures, BI-RADS categorization, HPE analysis, and the comparison of radiological findings with HPE results.

### 2.2. Imaging Protocols

DM and DBT were performed using a Selenia Dimensions FFDM system (Hologic, Bedford, MA, USA), equipped with a 70 μm selenium detector (detector size: 240 × 290 mm; pixel size: 70 μm). The system employed rhodium and silver filters with a tungsten X-ray tube. Standard projections included cranio-caudal (CC) and medio-lateral oblique (MLO) views, acquired at 25–49 kVp with a maximm tube current of 20 mA. DBT was performed bilaterally on the same device under identical compression and positioning. The X-ray tube rotated over an angular range of 15° in both CC and MLO views (25–49 kVp, maximum tube current of 200 mA, and aluminum filter). The selenium detector used for DBT had a size of 24 × 29 cm and a pixel size of 140 μm. Image reconstruction was completed with a 1 mm slice thickness and a reconstruction time of approximately 5 s. Supplemental breast US was conducted using a high-resolution ultrasound unit (Resona 7; Mindray, Shenzhen, China), equipped with an L14–4.5 MHz linear probe. Our study utilized handheld ultrasound (HHUS). All scans were conducted with the patient positioned in the supine and oblique supine positions to ensure adequate visualization of all quadrants and retro areolar regions of both breasts. The protocol included systematic scanning in both transverse and longitudinal planes, complete coverage of the breast tissue from the clavicle to the inframammary fold and from the sternum to the mid-axillary line, and lesion assessment based on the BI-RADS US lexicon, including evaluation of shape, orientation, margin, echo pattern, posterior features, and associated findings. The US readings were obtained from finalized institutional reports and were not reinterpreted or re-blinded for the study. The original US source images were not retrospectively reviewed, as a dedicated US image archive was not available.

Breast MRI was performed using a 3.0 T MRI unit (Siemens Medical Systems, Erlangen, Germany) with the patient in the prone position. Imaging was conducted using a dedicated 16-channel breast array coil and followed the institution’s standard imaging protocols. The protocol included a T2-weighted turbo spin-echo sequence in the axial plane, a T2-weighted STIR sequence in the sagittal plane, and diffusion-weighted imaging (DWI) with apparent diffusion coefficient (ADC) maps in the axial plane. These were followed by a dynamic contrast-enhanced (DCE) study, consisting of a total of seven T1-weighted 3D fast low-angle shot (FLASH) fat-saturated sequences acquired in the axial plane—one pre-contrast and six post-contrast acquisitions. Intravenous contrast was administered using a bolus injection of Gadobutrol (Gadovist, Bayer Schering Pharma, Berlin, Germany) at a dose of 0.1 mmol/kg body weight. Imaging parameters included a slice thickness of 2 mm and an acquisition matrix of 380 × 320, resulting in a voxel size of 0.4 × 0.4 × 2 mm. The temporal resolution for each dynamic acquisition was approximately 80 s. Post-processing steps included image subtraction and the generation of maximum intensity projections (MIPs). The morphological features and enhancement kinetics of lesions were analyzed. A detailed summary of the institutional MRI protocol parameters is presented in Table 1.

### 2.3. Image Interpretation

All images were interpreted independently for each modality—DM, DBT, and breast MRI—and subsequently evaluated in combination: DM + DBT, DM + DBT with US report, and DM + DBT + US with breast MRI. All imaging assessments were conducted according to the criteria outlined in the ACR BI-RADS atlas, 5th edition.

Breast density assessment was performed subjectively by radiologists, who visually estimated the relative amounts of dense and non-dense tissues on mammograms. Breasts containing sufficient dense tissue capable of obscuring small cancers were categorized as ACR C, while breasts with extremely dense tissue were categorized as ACR D.

All lesions identified on DM and DBT were evaluated based on their morphologic characteristics, including shape, margins, density, size, and the presence or type of calcifications. Lesions identified on breast MRI were evaluated for shape, margins, signal intensity, size, number of lesions, and kinetic characteristics such as dynamic contrast enhancement curves and enhancement patterns.

All findings were analyzed using BI-RADS scores from 1 to 5; BI-RADS category 0 was not permitted. BI-RADS categories 1, 2, and 3 were considered benign, while BI-RADS 4 (including 4A, 4B, and 4C) and 5 were considered suspicious for malignancy. Findings from US were obtained from our institutional database as final results, and source images were not available for review.

Finally, all imaging results were compared with HPE findings when available. Patients who did not undergo biopsy were followed with appropriate imaging for 12 to 24 months. If imaging findings remained stable over follow-up compared with previous examinations, those cases were ultimately considered benign.

The quantitative and qualitative analyses of the images were not blinded. Breast MRI images were interpreted by two experienced radiologists—one with 12 years and the other with 5 years of experience in breast MRI—who had access to the findings from the other three imaging modalities. DM, DBT, and US were independently interpreted by four radiologists with a knowledge of previous examination findings. Among them, there were two radiologists with 18 and 10 years of experience in interpreting DM, DBT, and US, while the other two had 5 years of experience in DM, DBT, and US. All radiologists were blinded to clinical and HPE data. DM and DBT images analyses and measurements were performed using a Clinical Picture Archiving And Communication System workstation monitor manufactured by SecurView workstations (Hologic) with a monitor resolution of 5 megapixels (MP) optimized to read both two-dimensional and three-dimensional images. MRI evaluation was conducted on a diagnostic workstation manufactured by Barco with a monitor resolution of 2 MP.

### 2.4. Statistical Methods

The analyzed group consisted of participants who were screened with all four modalities. The study sample size was 156 women. All four diagnostic methods were tested with Cochran’s K test and compared with the McNemar test. The sensitivity, specificity, positive and negative predictive value (PPV and NPV, respectively), and accuracy of the test were calculated based on the data obtained using Crosstabs tables. The chi-square test was used to calculate differences between categorical variables. Multivariate logistic regression was used to identify patient- or lesion-related factors that influenced diagnostic performance. Cancer diagnosis was based on the HPE of the biopsy sample. Statistical significance was assessed at *p* < 0.05. Statistical software analysis was performed using IBM SPSS Statistics, version 24.0 (Statistical Package for the Social Sciences—International Business Machines Corporation, Armonk, NY, USA).

## 3. Results

### 3.1. Patient Characteristics

**Figure 2:**
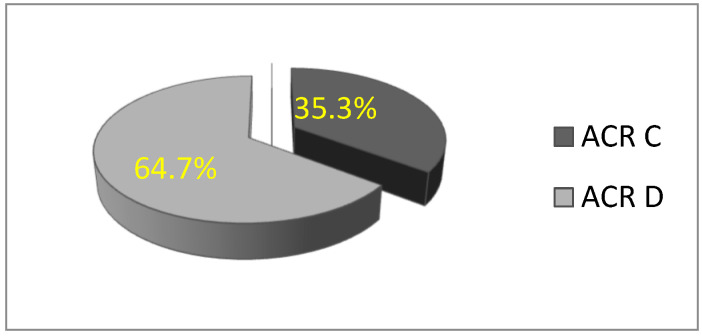
Distribution of women according to breast type: ACR C and D.

In this study, a total of 156 patients were included in the statistical analysis. All participants were women with dense breasts, with a mean age of 51.32 ± 9.82 years (age range: 28 to 77 years). The majority of women were premenopausal. Based on mammographic breast density classification, the rate of patients with ACR C breast density was 35.3% (n = 55), and 64.7% (*n* = 101) were ACR D, which is statistically significantly higher (χ^2^ = 13.564; df = 1; *p* = 0.000) (Figure 2).

### 3.2. Identification of Independent Predictors of Malignancy in Women with Dense Breasts

In order to identify independent predictors of malignancy in women with dense breasts, multivariate logistic regression analysis was performed. The following variables were included in the model: age, breast density (ACR), and imaging modality (US, DM, DBT, and breast MRI) (Table 2).

A multivariate logistic regression analysis was conducted to evaluate the independent contribution of patient age, breast density (ACR), and imaging modality(DM, DBT, US, and breast MRI) to predicting malignancy. The analysis showed that age (OR = 0.235; 95% CI: 0.002–24.434; *p* = 0.541) and breast density (ACR category) (OR = 1.640; 95% CI: 0.093–28.771; *p* = 0.735) were not statistically significant predictors of malignancy. Similarly, ultrasound (US) did not have a significant effect (OR = 0.137; 95% CI: 0.002–8.880; *p* = 0.350). DM and DBT were also not statistically significant, with wide confidence intervals indicating instability in estimates (DM: OR = 1.052; 95% CI: 0.000–59,270.318; *p* = 0.993; DBT: OR = 0.377; 95% CI: 0.000–18128.955; *p* = 0.859). Importantly, breast MRI emerged as the only statistically significant independent predictor of malignancy. The odds of malignancy were significantly lower when breast MRI was negative (OR = 0.005; 95% CI: 0.000–0.089; *p* < 0.001), indicating its strong diagnostic value in identifying malignant lesions.

**Figure 3:**
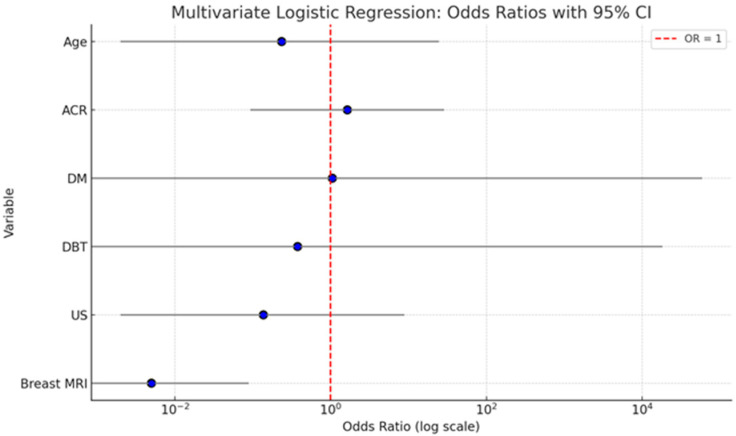
Here is a summary graph displaying the odds ratios (ORs) and 95% confidence intervals for each variable in the multivariate logistic regression model. The red dashed line at OR = 1 indicates the threshold of no effect.

The summary graph (Figure 3) illustrates the odds ratios (ORs) with 95% confidence intervals (CIs) for each variable included in the multivariate logistic regression model assessing predictors of malignancy. Breast MRI stands out as the only statistically significant predictor. Its OR is substantially below 1 (OR = 0.005), with a narrow CI (0.000–0.089), and the entire confidence interval lies well below the reference line at OR = 1. This indicates a strong inverse association: when breast MRI is negative, the probability of malignancy is extremely low, underscoring its high diagnostic accuracy. Age and ACR show ORs slightly above or below 1, but their CIs are wide and cross the reference line, reflecting substantial uncertainty and non-significant *p*-values. This suggests that neither factor independently predicts malignancy in the adjusted model. DM and DBT both show very wide confidence intervals with ORs close to 1, indicating imprecise estimates and no significant contribution to the model when adjusted for other variables. US also demonstrates a non-significant association with malignancy, with an OR < 1 but a wide CI that spans the null value.

Table 3 shows diagnostic performance stratified by age, menopausal status, and lesion type (invasive cancer vs. DCIS).

The average age of patients with malignant tumors was 52.6 ± 11.1 years, compared with 49.9 ± 8.1 years for those with benign lesions. Although patients with malignant disease tended to be older, this difference was not statistically significant (t(154) = −1.726; *p* = 0.086). No statistically significant association was found between menopausal status and pathological findings, nor between pre- and postmenopausal statuses in relation to malignancy. Notably, all 12 cases of ductal carcinoma in situ (DCIS) were observed exclusively within the subgroup of patients with malignant tumors.

### 3.3. Analysis of HPE Findings of Needle/Surgical Biopsies

Out of the total 156 patients, 120 breast lesions were HPE-verified. The detailed distribution of HPE diagnoses is presented in Table 2. The remaining 36 women were followed up at predefined intervals and were ultimately categorized as having benign breast findings based on imaging stability over time.

According to Table 4, 101/120 (84.2%) were HPE-confirmed as malignant, while 19/120 (15.8%) were HPE-confirmed as benign. Among the malignant tumors, 80 cases (79.2%) were diagnosed as invasive carcinoma and 21 cases (20.8%) as carcinoma in situ. Of the 80 invasive cancers, the most common histological subtype was invasive ductal carcinoma, accounting for 61 cases (76.2%), followed by invasive lobular carcinoma in 12 (15%), papillary carcinoma in 3 (3.75%), mucinous carcinoma in 2 (2.5%), and breast metastases in 2 cases (2.5%).

The majority of benign lesions were identified as fibroadenomas (47.3%) and fibrocystic changes (31.5%).

### 3.4. Statistical Analysis of Four Radiological Diagnostics—US, DM, DBT and Breast MRI—In Correlation with HPE Results

As presented in Table 5, the majority of detected lesions across all four imaging modalities were classified as BI-RADS 5, indicating a high suspicion of malignancy, comprising 37% (*n* = 231/624) of all categorized lesions. Suspicious lesions categorized as BI-RADS 4 (including subcategories 4A, 4B, and 4C) were more frequently associated with benign HPE outcomes—accounting for 59% (*n* = 111/188) of benign lesions—compared with 41% (*n* = 77/188) in malignant cases.

The combination of DM and DBT reduced the number of false-negative findings but resulted in an increase in false positives. When US was added, false-negative findings decreased further from 8 to 3, while false positives increased from 40 to 51. The additional inclusion of breast MRI further reduced the false negatives to 2 but increased the false positives slightly to 53.

The highest concordance between imaging findings and HPE was observed with MRI (136/156; 87.2%), while the lowest concordance was recorded for DM (108/156; 69.2%).

According to Table 6, the number of false-negative findings varied across modalities: MRI (*n* = 4), US (*n* = 8), DBT (*n* = 9), and DM (*n* = 10). In contrast, US had the highest number of false-positive findings (*n* = 39), while MRI had the fewest (*n* = 16). Statistical analysis using the McNemar test revealed a statistically significant difference in cancer detection across all four imaging modalities in relation to HPE findings (*p* < 0.001) (Table 6). Similarly, Cochran’s Q test confirmed a significant difference in diagnostic performance among the four methods (Q = 12.101; df = 3; *p* = 0.007).

The sensitivity, specificity, positive predictive value (PPV), negative predictive value (NPV), and diagnostic accuracy of each diagnostic method in assessing the detected lesions are presented in Table 7.

DM demonstrated the lowest sensitivity (87.7%) and low specificity (49.3%) among the diagnostic methods evaluated for dense breasts. The combination of DM + DBT + US yielded a higher sensitivity (96.3%) compared with the combination of DM + DBT (90.1%), or the use of DM, DBT, and US alone (87.7%, 88.9%, and 90.1%, respectively). However, this combination showed a lower specificity of 32%, compared with 46.7% for DM + DBT, and 48%, 49.3%, and 56% for US, DM, and DBT used individually, respectively.

Adding MRI increased the sensitivity to 97.5% but resulted in a further decrease in specificity to 29.3%. MRI alone achieved a high sensitivity of 95.1% and the highest specificity of 78.7% among all individually used diagnostic methods.

Our findings indicate that breast MRI provided the highest positive predictive value (PPV) and negative predictive value (NPV) for dense breasts, 82.8% and 93.7%, respectively. In contrast, DM had the lowest PPV (65.1%) and NPV (78.7%). When all four imaging modalities were combined, the sensitivity reached 97.5%, but the specificity dropped significantly to 29.3%. Among all diagnostic approaches, breast MRI alone yielded the highest diagnostic accuracy (87.2%), while the combination of all four modalities had the lowest accuracy (64.7%).

**Figure 4:**
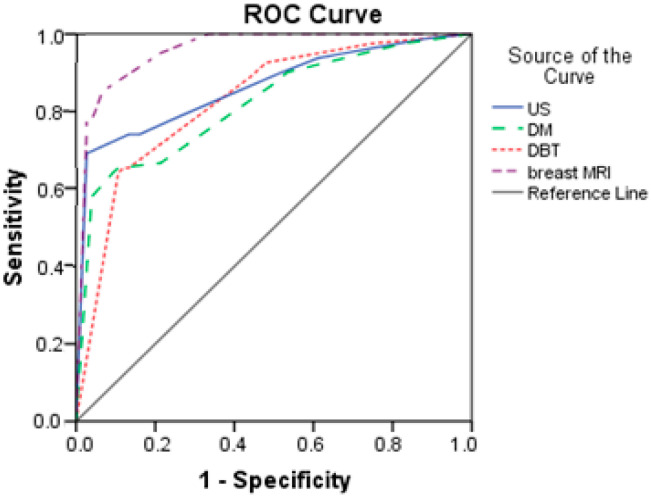
Comparison of ROC curves for US, DM, DBT, and breast MRI.

Based on the results from Table 7 and Figure 4, breast MRI had excellent predictive power (AUC = 0.958; SE = 0.015). The other three diagnostics methods had good prediction, with AUCs higher than 0.800. DM had the lowest discrimination power with an AUC of 0.820.

A detailed subgroup analysis of diagnostic performance stratified by breast density of ACR C versus ACR D is presented in Table 8.

US demonstrated slightly higher sensitivity in the ACR D group (75.2%) compared with the ACR C group (65.5%), likely reflecting its superior performance in dense tissue relative to DM. DM showed reduced sensitivity in the ACR D group (66.3% versus 76.4% in the ACR C group), as expected due to the masking effect of dense fibro glandular tissue on tumor visibility. DBT and breast MRI exhibited comparable diagnostic performance across both density categories (DBT: 67.3% in both groups; MRI: 58.2% in ACR C versus 60.4% in ACR D), indicating their stability and reliability regardless of breast density.

### 3.5. Selected Interesting Examples from Our Study

**Figure 5:**
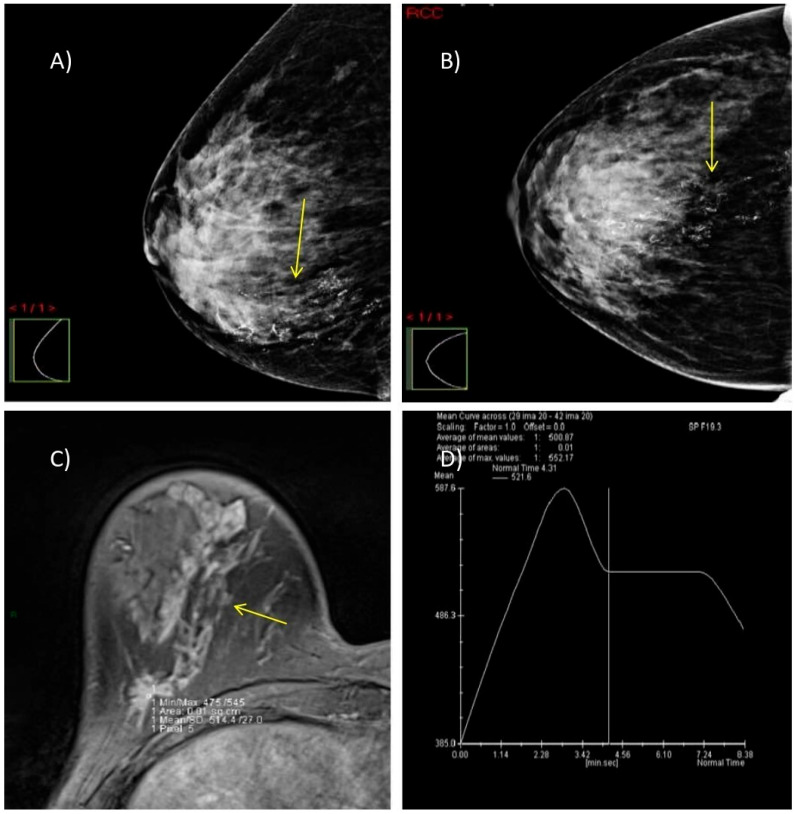
A case of a 47-year-old woman with dense breasts (ACR category C) and HPE-verified multifocal invasive ductal carcinoma, grade 2 in the right breast. (**A**,**B**) DM in MLO and CC projections shows highly suspicious microcalcifications with segmental distribution at the junction of the lower breast quadrants (yellow arrow). (**C**) Axial 3D T1-weighted FLASH fat-suppressed MR image demonstrates an extensive post-contrast hyperintense mass lesion extending from the nipple to the prepectoral space (yellow arrow). (**D**) MRI dynamic contrast enhancement curve type III (“wash out” curve).

**Figure 6:**
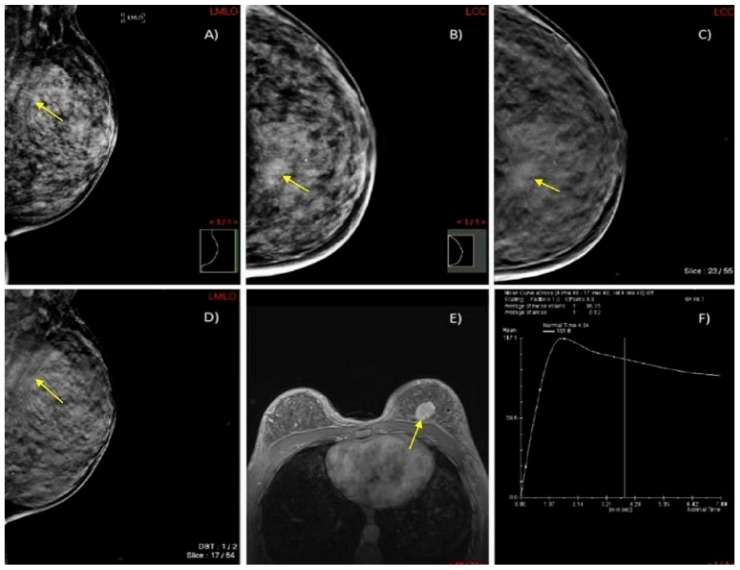
A case of a 45-year-old woman with extremely dense breasts (ACR category D) and HPE-confirmed invasive ductal cancer in the left breast. (**A**,**B**) DM in MLO and CC projections shows an architectural distortion in the upper inner quadrant of the left breast (yellow arrow). (**C**,**D**) DBT in MLO and CC projections illustrates a spiculate mass in the upper inner quadrant of the left breast (yellow arrow). (**E**) Axial 3D T1-weighted FLASH fat-suppressed (FS) MR image demonstrates an extensive post-contrast hyperintense lesion in the prepectoral space of the left breast (yellow arrow). (**F**) MRI dynamic contrast enhancement curve type III (“wash out” curve).

We present two illustrative cases from our female patient cohort, each with HPE-confirmed diagnosis in dense breasts. These lesions were identified using MRI, DM, and DBT, as demonstrated in Figure 5 and Figure 6.

## 4. Discussion

In breast imaging, recent studies have demonstrated that women with dense breast tissue are often underdiagnosed when DM is used as the only screening modality. The adequacy of DBT alone in women with extremely dense breasts (ACR D), as well as the added value of supplemental US, remains uncertain and warrants further investigation. This highlights the ongoing need to refine diagnostic algorithms for this population to improve early breast cancer detection and reduce the incidence of interval cancers.

While numerous studies have evaluated individual or paired imaging modalities for breast cancer detection in women with dense breast tissue, few have undertaken direct comparisons across multiple imaging methods within the same patient cohort. Prior comparative studies have typically focused on specific combinations, such as DM versus DBT, DM + DBT versus US, or DM + DBT versus MRI. These investigations have provided valuable insights into the incremental benefits of supplemental imaging but have not simultaneously assessed all four modalities within a unified diagnostic pathway. To the best of our knowledge, our study is among the first to retrospectively compare DM, DBT, US, and breast MRI applied within the same cohort of women with dense breasts, offering a comprehensive multimodal evaluation in a real-world diagnostic setting. This approach provides a rare opportunity to assess the relative and combined diagnostic performance of these modalities while highlighting their complementary strengths and trade-offs.

In our study, we evaluated and compared the diagnostic performance of four imaging modalities—DM, DBT, US, and breast MRI—in a cohort of 156 women with dense breasts. Receiver operating characteristic (ROC) curve analysis revealed that the combination of DM and DBT, when supplemented with US, achieved predictive accuracy comparable to that of breast MRI. This finding is particularly relevant in clinical settings where MRI is less accessible, as these alternative modalities are more widely available and more easily integrated into routine screening workflows.

However, we also observed a notable decline in specificity when combining multiple imaging techniques—especially with the full combination of DM + DBT + US + MRI—which raises concerns about overdiagnosis. Such a decline may lead to a higher number of false positives, resulting in unnecessary biopsies, increased psychological distress and anxiety for patients, and a greater burden on healthcare systems in terms of cost and resource allocation. These consequences may, in some cases, offset the benefits of improved sensitivity. Therefore, an optimal balance between diagnostic sensitivity and specificity is essential to ensure that breast cancer screening strategies are both effective and sustainable.

In this study, age appeared to trend toward a higher mean in patients with malignant lesions compared with those with benign findings (52.6 vs. 49.9 years), although this difference did not reach statistical significance (*p* = 0.086). While not conclusive, this observation aligns with the general understanding that breast cancer risk increases with age. Menopausal status, evaluated both through chi-square tests of independence and goodness-of-fit tests, showed no significant association with malignancy, indicating that menopausal transition alone may not be a distinguishing factor in this cohort. As expected, all invasive cancers and ductal carcinoma in situ (DCIS) cases were confirmed as malignant on histopathological examination, reinforcing their classification within the malignant spectrum. These findings underscore the complexity of age and hormonal status as risk factors, suggesting that while they may contribute to overall risk stratification, they may not independently predict malignancy in diagnostically ambiguous cases.

Multivariate logistic regression analysis revealed that among the examined factors—age, breast density (ACR), and imaging modalities—only breast MRI was a statistically significant independent predictor of malignancy. While age showed a slight trend toward increased risk with advancing years (OR = 1.032; 95% CI: 0.998–1.068; *p* = 0.064), this association did not reach statistical significance. Similarly, breast density classified as ACR D did not independently predict malignancy (OR = 1.480; 95% CI: 0.753–2.910; *p* = 0.256), highlighting that dense breast tissue alone may not be sufficient for malignancy risk stratification in this cohort. Among imaging modalities, digital mammography (DM), digital breast tomosynthesis (DBT), and ultrasound (US) were not statistically significant predictors, with notably wide confidence intervals reflecting high variability and reduced estimate precision. In contrast, breast MRI demonstrated a strong and statistically significant inverse association with malignancy when negative (OR = 0.005; 95% CI: 0.000–0.089; *p* < 0.001), reinforcing its high negative predictive value and diagnostic reliability. These findings underscore the central role of breast MRI in the accurate identification of malignant lesions, even after adjusting for patient-related and modality-related factors.

DM alone had the lowest sensitivity and low specificity in women with dense breast tissue, consistent with the results of other studies. The addition of DBT to DM resulted in an increased number of detected lesions compared with either modality used individually and therefore improved sensitivity and NPV (87.7% and 78.7% to 90.1% and 81.4%, respectively). This improvement can be attributed to the reduction in tissue overlap provided by DBT, which enhances lesion visibility in dense breast tissue. However, this combination led to a slight decrease in specificity and positive predictive value (PPV) likely due to a higher rate of false-positive findings (from 49.3% and 65.1% to 46.7% and 64.6%, respectively), possibly resulting from factors such as suboptimal technical positioning, perception errors, and lesion characteristics.

Mousa et al. also assessed the added value of DBT when combined with DM in the detection and characterization of various breast lesions in dense breasts. Their study showed that the sensitivity, specificity, and accuracy of DM significantly improved with the addition of DBT [37]. These findings are consistent with those reported in previous studies by Skaane, Østerås, and Hassan [38,39,40]. Studies show that DBT improves both sensitivity and specificity compared with DM, particularly for masses and architectural distortions that are otherwise obscured in dense tissue [41].

A study published by Sudhir et al. compared contrast-enhanced digital mammography (CEDM) with synthetic DM, DBT, and US in the evaluation of 166 lesions in 130 female patients with dense breasts. The authors reported that synthetic DM had the lowest sensitivity, specificity, PPV, and accuracy; in contrast, DBT demonstrated higher sensitivity and specificity. Notably, CEDM achieved sensitivity, specificity, and accuracy that are comparable to our findings for breast MRI. These results highlight the potential of CEDM as an effective alternative to breast MRI, particularly in settings where MRI availability is limited. Further research at our institution is warranted to explore the clinical utility of CEDM and its comparability to breast MRI in the assessment of dense breast tissue [42].

In the study conducted by Ha et al., the supplemental US after negative findings on DBT or DM improved detecting breast cancer in women with dense breasts compared with the DM and DBT alone [21]. Similarly, our study found that adding US to DM and DBT increased sensitivity to 96.3%. However, this improvement in sensitivity was accompanied by a decrease in specificity and overall accuracy, which dropped to 32% and 65.4%, respectively. The enhanced sensitivity of this three-modality approach, compared with DM alone, is consistent with findings reported by Mariscotti et al. [43].

US suffers from operator dependence and reduced specificity, which can lead to higher false-positive rates, as well as difficulty in detecting microcalcifications [24]. Automated breast ultrasound (ABUS) seeks to address these limitations, notably improving lesion detection rates when combined with mammography, showing increased sensitivity compared with mammography alone, but still faces technological constraints [44]. Emerging innovations like B7-H3-targeted molecular ultrasound imaging further boost diagnostic accuracy by leveraging tumor-specific vascular markers, suggesting future potential for highly personalized, precise screening protocols [45]. These advancements collectively affirm US’s growing role in improving breast cancer detection, particularly in populations underserved by standard DM, and support the trend toward multimodal, patient-tailored diagnostic strategies.

In our study, the diagnostic agreement with HPE results was 69.2% for DM, 69.9% for US, 73.1% for DBT, and 87.2% for breast MRI. Breast MRI demonstrated the highest concordance, consistently with prior studies, such as that by Pereira et al., who reported agreement rates of 71.9% for DM, 46.9% for US, and 75% for breast MRI [46]. This highlights breast MRI’s superior agreement with HPE results while also reflecting variability in US performance, potentially due to operator dependency or lesion characteristics.

Meta-analyses consistently confirm breast MRI’s superior performance, particularly in women with genetic predispositions to breast cancer [47]. Reviews report MRI sensitivity as high as 94.6%, significantly outperforming US and DM in dense breast tissue [48]. Our study’s MRI sensitivity (95.3%) and specificity (78.2%) are consistent with these reports. Recent advances, such as abbreviated breast MRI (AB-MRI) protocols, improve feasibility, reducing acquisition and interpretation times while maintaining diagnostic performance.

Notable differences in diagnostic performance were observed across breast density categories; nevertheless, there was the methodological limitation imposed by selective biopsy of only suspicious lesions. Ultrasound demonstrated higher detection rates of malignancy in the ACR D group, suggesting improved efficacy in dense breast tissue. In contrast, digital mammography showed reduced performance in the same group, likely due to the masking effect of dense parenchyma. The stable diagnostic yield of DBT across both ACR C and D categories highlights its robustness and reinforces its utility in dense breasts. MRI exhibited consistent performance irrespective of breast density, aligning with its role as the imaging modality least influenced by tissue composition.

Despite its diagnostic advantages, breast MRI’s adoption is constrained by economic and logistical barriers, including high cost, limited MRI unit availability, longer acquisition and interpretation times, and a shortage of trained personnel. Such constraints are particularly pronounced in low-resource settings, where infrastructure limitations can result in long waiting lists and delayed diagnoses. These practical challenges must be carefully weighed when considering MRI as a screening tool for women with dense breasts. Future research should focus on cost-effectiveness analyses and scalable strategies to ensure equitable access.

Emerging approaches such as abbreviated MRI (AB-MRI) protocols and artificial intelligence (AI)-assisted image interpretation offer promise for overcoming some of the logistical and economic barriers, improving efficiency and access while maintaining diagnostic quality. In recent meta-analyses, AB-MRI showed pooled sensitivity and specificity rates comparable to full protocols [49]. This streamlined approach may facilitate broader implementation in screening programs, particularly in resource-limited settings where scanner availability and patient throughput are critical constraints. In parallel, AI-assisted diagnostic tools are being developed to improve lesion detection and shorten interpretation times, thereby supporting radiologists in managing increasing imaging volumes. Together, these innovations may help bridge the gap between MRI’s recognized diagnostic value and its current limited accessibility in routine breast cancer screening, particularly for women with dense breasts. The integration of AI into the interpretation of DM and DBT should also be explored, as AI has the potential to reduce perception errors and improve diagnostic performance by lowering both false-negative and false-positive rates. Emerging AI-based tools can assist radiologists by providing more standardized assessments and reducing interobserver variability. This could be particularly valuable in dense breast imaging, where conventional methods face significant limitations.

This study has several limitations. It was conducted at a single academic center with a relatively small sample size (*n* = 156), which limits its generalizability. Breast composition was assessed in a way that introduced potential interobserver variability. The lack of objective quantification methods, such as volumetric or automated density assessment tools, further limits the precision and consistency of density categorization. In recent years, there has been increasing emphasis on using objective, automated methods for evaluating breast density, which may provide more consistent and reproducible results. Image interpretation was not blinded, and the radiologists who evaluated breast MRI had access to the findings from DM, DBT, and US. This could have introduced verification bias, potentially inflating the diagnostic performance of MRI. While this reflects real-world clinical practice—where radiologists often interpret imaging in the context of previous results—it may have overestimated MRI’s independent accuracy. Future studies should consider a blinded, modality-specific assessment protocol to objectively compare each method and isolate their individual contributions to diagnostic performance. As the study was conducted at a tertiary care cancer center, the inclusion of both symptomatic and asymptomatic patients within the cohort led to a patient selection bias and a higher prevalence of malignancy than in general screening populations, which affects the reported diagnostic performance metrics, particularly sensitivity and positive predictive value. This could have potentially overestimated diagnostic performance. The absence of retrospective US image review and interobserver variability assessment represents an additional limitation given US’s operator dependence. Future studies should aim for multicenter, prospective designs with larger and diverse patient populations, blinded image interpretation, and objective breast density assessment to validate and generalize these findings.

## 5. Conclusions

To the best of our knowledge, no studies in the literature have examined the diagnostic performance of all four imaging modalities in women with dense breasts. Our study makes a unique contribution by simultaneously evaluating all four methods, providing a rare and comprehensive comparison that is essential to optimizing protocols in this population. The findings support the strategic integration of complementary modalities to improve early breast cancer detection, offering the highest predictive value while aiming to minimize false negatives and limit unnecessary invasive procedures. Despite certain limitations, our results underscore the benefits of early cancer detection in dense breasts where improved outcomes tend to outweigh the disadvantages, reinforcing current recommendations for their use. Specifically, our findings confirm the significant diagnostic improvement that DBT offers over DM alone due to superior tissue differentiation. We also underline the value of US in dense breast tissue, despite its relatively high false-positive rate. Breast MRI, when used independently, affirms its role as the gold standard in dense breast imaging, although its broader application is limited by cost and accessibility. In settings where MRI is not feasible, our results support a substitution strategy utilizing DM + DBT + US while also emphasizing the need for caution to avoid overreliance due to specificity concerns.

## Figures and Tables

**Table 1:** The parameters of MRI sequence acquisition.

Sequence	T2W TSE	T2W STIR	DWI (EPI)	T1W 3D FLASH FS Before and After Bolus Injection of Contrast Media
Imaging plane	Axial	Sagittal	Axial	Axial
TR/TE (ms) (time to repeat/time to echo)	4900/76	6800/70	3700/60/80	5/3
FA (°) (flip angle)	120	120	150	10
FOV (mm) (field of view)	340	240	340	340
Matrix size	380 × 380	320 × 290	220/220	380 × 320
Slice thickness (mm)	2	4	4	2
Number of slices	60	40	30	80
Voxel size (mm)	1 × 1 × 2	0.7 × 0.7 × 4	1.5 × 1.5 × 4	0.4 × 0.4 × 2
Time of acquisition (s)/number of acquisitions	120/2	120/2	180/1	80/7
B value (s/mm^2^)	-	-	50; 400; 800	-
Scan time (s)	222	206	179	592

**Table 2:** Results of multivariate logistic regression: influence of age, breast density, and imaging modality (US, DM, DBT, and breast MRI) on diagnostic performance.

	B	S.E.	Wald	df	Sig.	Exp (B)	95% C.I. for EXP (B)	
							Lower	Upper
age	−1.446	2.369	0.373	1	0.541	0.235	0.002	24.434
ACR	0.495	1.462	0.114	1	0.735	1.640	0.093	28.771
DM	0.051	5.581	0.000	1	0.993	1.052	0.000	59,270.318
DBT	−0.976	5.501	0.031	1	0.859	0.377	0.000	18,128.955
US	−1.988	2.128	0.872	1	0.350	0.137	0.002	8.880
Breast MRI	−5.226	1.429	13.365	1	0.000	0.005	0.000	0.089
Constant	4.472	1.551	8.311	1	0.004	87.543		

Abbreviations: B: regression coefficient; S.E.: standard error; df: degrees of freedom; Sig: significance level; Exp: odds ratio; ACR: American College of Radiology breast density type; DM: digital mammography; DBT: digital breast tomosynthesis; US: ultrasound; MRI: magnetic resonance imaging.

**Table 3:** Stratification by age, menopause, and invasive cancer vs. DCIS lesion type in relation to HPE.

	Yes	No	Significance
Age (years) ^#^	52.6 (11.1)	49.9 (8.1)	0.086
Menopause ^$^			0.906
Yes ^&^	45 (55.6)	36 (44.4)	0.317
No ^&^	40 (53.3)	35 (46.7)	0.564
DCIS	9 (100)	0 (0.0)	

Abbreviations: ^#^: *t*-test of independence; mean (standard deviation). ^$^: chi-square test of independence. ^&^: chi-square goodness-of-fit test; number (percentile). DCIS: ductal cancer in situ.

**Table 4:** HPE diagnoses of benign and malignant lesions.

HPE Finding	Benign Lesions	Malignant Lesions
	*n*	*n*
Invasive ductal carcinoma	0	61
Invasive lobular carcinoma	0	12
DCIS	0	17
LCIS	0	3
Papillary carcinoma	0	3
PCIS	0	1
Mucinous carcinoma	0	2
Metastases	0	2
Fibroadenoma	9	0
Fibrocystic breast changes (FCC)	6	0
Adenosis	1	0
Granulomatous mastitis	1	0
Papilloma	1	0
Mucocele-like lesions	1	0
TOTAL	19	101

Abbreviations: HPE: histopathological examination; DCIS: ductal carcinoma in situ; LCIS: lobular carcinoma in situ; PCIS: papillary carcinoma in situ.

**Table 5:** Correlation of HPE findings and BI-RADS categories for all four diagnostics.

BI-RADS Category	US		DM		DBT		Breast MRI	
	Malignant	Benign	Malignant	Benign	Malignant	Benign	Malignant	Benign
1	1	9	2	13	2	19	0	14
2	4	20	6	22	4	20	0	36
3	3	7	2	2	3	3	4	9
4	10	21	17	22	19	23	8	11
4A	3	6	1	8	0	0	6	2
4B	0	2	1	1	0	1	0	1
4C	4	8	5	4	1	1	2	0
5	56	2	47	3	52	8	61	2

Abbreviations: BI-RADS: Breast Imaging Reporting and Data System; US: ultrasound; DM: digital mammography; DBT: digital breast tomosynthesis; MRI: magnetic resonance imaging.

**Table 6:** Pooled analysis of US, DM, DBT, and breast MRI results and comparison with HPE diagnosis.

Type of Lesion	Histopathology			
	Malignant	Benign	Total	McNemar Test
US				*p* < 0.001
Malignant	73 (TP)	39 (FP)	112	
Benign	8 (FN)	36 (TN)	44	
DM				*p* < 0.001
Malignant	71 (TP)	38 (FP)	109	
Benign	10 (FN)	37 (TN)	47	
DBT				*p* < 0.001
Malignant	72 (TP)	33 (FP)	105	
Benign	9 (FN)	42 (TN)	51	
Breast MRI				*p* < 0.001
Malignant	77 (TP)	16 (FP)	93	
Benign	4 (FN)	59 (TN)	63	
DM + US				*p* < 0.001
Malignant	78 (TP)	50 (FP)	128	
Benign	3 (FN)	25 (TN)	28	
DM + DBT				*p* < 0.001
Malignant	73 (TP)	40 (FP)	113	
Benign	8 (FN)	35 (TN)	43	
DM + DBT + US				*p* < 0.001
Malignant	78 (TP)	51 (FP)	129	
Benign	3 (FN)	24 (TN)	27	
DM + DBT + US with breast MRI				*p* < 0.001
Malignant	79 (TP)	53 (FP)	132	
Benign	2 (FN)	22 (TN)	24	
Total	81	75	156	

Abbreviations: US: ultrasound; DM: digital mammography; DBT: digital breast tomosynthesis; MRI: magnetic resonance imaging; TP: true positive; FP: false positive; TN: true negative; FN: false negative.

**Table 7:** Diagnostic performance of DM, DBT, US, and breast MRI in lesion detection in all 156 examined women.

	Sensitivity	Specificity	PPV	NPV	Overall Accuracy
US	0.901	0.480	0.652	0.818	0.699
DM	0.877	0.493	0.651	0.787	0.692
DBT	0.889	0.560	0.686	0.824	0.731
Breast MRI	0.951	0.787	0.828	0.937	0.872
DM + DBT	0.901	0.467	0.646	0.814	0.692
DM + DBT + US	0.963	0.320	0.605	0.889	0.654
DM + DBT + US + MRI	0.975	0.293	0.598	0.917	0.647

Abbreviations: PPV: positive predictive value; NPV: negative predictive value; US: ultrasound; DM: digital mammography; DBT: digital breast tomosynthesis; MRI: magnetic resonance imaging.

**Table 8:** Diagnostic performance stratified by breast density of ACR C versus ACR D.

	ACR C (*n* = 55)		ACR D (*n* = 101)	
	Malignancy	No Malignancy	Malignancy	No Malignancy
US	36 (65.5%)	19 (34.5%)	76 (75.2%)	25 (24.8%)
DM	42 (76.4%)	13 (23.6%)	67 (66.3%)	34 (33.7%)
DBT	37 (67.3%)	18 (32.7%)	68 (67.3%)	33 (32.7%)
Breast MRI	32 (58.2%)	23 (41.8%)	61 (60.4%)	40 (39.6%)

Abbreviations: US: ultrasound; DM: digital mammography; DBT: digital breast tomosynthesis; MRI: magnetic resonance imaging.

## Data Availability

The data that support the finding of this study are available from the corresponding author (D.P.) upon reasonable request.
